# Accuracy of genome-wide imputation of untyped markers and impacts on statistical power for association studies

**DOI:** 10.1186/1471-2156-10-27

**Published:** 2009-06-16

**Authors:** Ke Hao, Eugene Chudin, Joshua McElwee, Eric E Schadt

**Affiliations:** 1 Genetics Department, Rosetta Inpharmatics, a Wholly Owned Subsidiary of Merck & Co Inc, Seattle, Washington, USA

## Abstract

**Background:**

Although high-throughput genotyping arrays have made whole-genome association studies (WGAS) feasible, only a small proportion of SNPs in the human genome are actually surveyed in such studies. In addition, various SNP arrays assay different sets of SNPs, which leads to challenges in comparing results and merging data for meta-analyses. Genome-wide imputation of untyped markers allows us to address these issues in a direct fashion.

**Methods:**

384 Caucasian American liver donors were genotyped using Illumina 650Y (Ilmn650Y) arrays, from which we also derived genotypes from the Ilmn317K array. On these data, we compared two imputation methods: MACH and BEAGLE. We imputed 2.5 million HapMap Release22 SNPs, and conducted GWAS on ~40,000 liver mRNA expression traits (eQTL analysis). In addition, 200 Caucasian American and 200 African American subjects were genotyped using the Affymetrix 500 K array plus a custom 164 K fill-in chip. We then imputed the HapMap SNPs and quantified the accuracy by randomly masking observed SNPs.

**Results:**

MACH and BEAGLE perform similarly with respect to imputation accuracy. The Ilmn650Y results in excellent imputation performance, and it outperforms Affx500K or Ilmn317K sets. For Caucasian Americans, 90% of the HapMap SNPs were imputed at 98% accuracy. As expected, imputation of poorly tagged SNPs (untyped SNPs in weak LD with typed markers) was not as successful. It was more challenging to impute genotypes in the African American population, given (1) shorter LD blocks and (2) admixture with Caucasian populations in this population. To address issue (2), we pooled HapMap CEU and YRI data as an imputation reference set, which greatly improved overall performance. The approximate 40,000 phenotypes scored in these populations provide a path to determine empirically how the power to detect associations is affected by the imputation procedures. That is, at a fixed false discovery rate, the number of cis-eQTL discoveries detected by various methods can be interpreted as their relative statistical power in the GWAS. In this study, we find that imputation offer modest additional power (by 4%) on top of either Ilmn317K or Ilmn650Y, much less than the power gain from Ilmn317K to Ilmn650Y (13%).

**Conclusion:**

Current algorithms can accurately impute genotypes for untyped markers, which enables researchers to pool data between studies conducted using different SNP sets. While genotyping itself results in a small error rate (e.g. 0.5%), imputing genotypes is surprisingly accurate. We found that dense marker sets (e.g. Ilmn650Y) outperform sparser ones (e.g. Ilmn317K) in terms of imputation yield and accuracy. We also noticed it was harder to impute genotypes for African American samples, partially due to population admixture, although using a pooled reference boosts performance. Interestingly, GWAS carried out using imputed genotypes only slightly increased power on top of assayed SNPs. The reason is likely due to adding more markers via imputation only results in modest gain in genetic coverage, but worsens the multiple testing penalties. Furthermore, cis-eQTL mapping using dense SNP set derived from imputation achieves great resolution, and locate associate peak closer to causal variants than conventional approach.

## Background

It has been estimated that the human genome contains 7.5 million common single nucleotide polymorphisms (SNPs) with minor allele frequencies (MAF) ≥ 5% [1], and only a fraction of these (e.g. hundreds of thousands of SNPs) can be directly assayed using current high-density microarrays. Because of linkage disequilibrium (LD) among nearby markers, many untyped SNPs are highly correlated with one or more nearby assayed SNPs. Therefore, testing assayed SNPs for association to traits of interest will also have some power to capture signals for untyped causal SNPs. Further, if the assayed SNPs are strategically distributed across the genome (e.g. tag SNPs), maximal genetic coverage can be achieved [1-3]. Typical genetic association studies examine assayed SNPs for association with phenotypes, where significant signals suggest causal SNPs in the surveyed region. To enhance this type of analysis, the genotypes of unobserved SNPs can be imputed (i.e., predicted) based on nearby markers and then directly tested for association with phenotypes of interest [4-6]. This strategy has several advantages: (1) allows researchers to directly combine experiments carried out on different microarrays (e.g. Illumina and Affymetrix arrays) for meta-analyses; (2) enables researchers to easily replicate/compare previous finding across array types; and (3) enables testing on a large number of SNPs to reveal the fine structure of the association peak, facilitating interpretation of results and location of the causal polymorphisms.

At present, the performance of genome-wide imputation (GWI) of SNP genotypes has not been systematically quantified in the context of a genome-wide association study (GWAS), and GWI's impact on statistical power in the context of GWAS is not fully understood. In this paper, we systematically benchmark the yield and accuracy of GWI and the influence a number of factors, including genotyping arrays, ethnicity, reference panel, and LD structure, on GWI performance. Further, we leverage large-scale empirical data to investigate whether incorporating GWI data in GWAS will result in additional statistical power and will enhance ability to position the association peak closer to causal variants.

## Results

Several GWI methods have been developed [5-12], and previous studies have well documented the accuracy of these methods with respect to imputing missing genotypes, as well as untyped SNPs. We find BEAGLE [11] and MACH [7] algorithms are able to impute missing genotypes with high accuracy. On our dataset, MACH slightly outperformed BEAGLE in imputing missing genotypes (Additional file 1). These results are consistent with intensive comparison on existing imputation methods [11,13], including MACH, BEAGLE, IMPUTE, fastPHASE and PLINK. These methods perform quite similarly although MACH and IMPUTE are the best in term of accuracy. We adopted the MACH algorithm for all subsequent analyses, and mainly focus on imputing markers that are totally untyped.

### Imputation yield and accuracy

We employed the CATIE (Clinical Antipsychotic Trials of Intervention Effectiveness) and an expanded version of the deLiver study datasets [14,15], because these cohorts have several appealing characteristics. First, both studies contain Caucasian and African American subjects. Second, both studies utilized advanced microarray products: samples in CATIE were genotyped using the Affymetrix 500 K + 164 K custom array and deLiver samples were genotyped using the Illumina HumanHap 650Y (Ilmn650Y). From Ilmn650Y, Illumina HumanHap 317 K (Ilmn317K) data can be derived (see materials and methods). Finally, approximately 40,000 mRNA expression traits were measured on the deLiver cohort, providing a unique opportunity to assess statistical power empirically for GWAS when incorporating WGI data.

**Figure 1:**
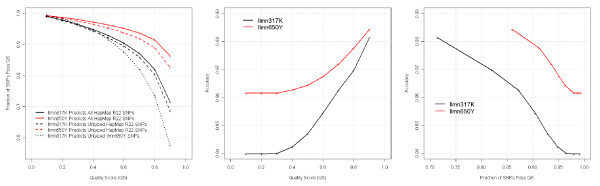
**Genome-wide imputation based on the Ilmn650Y data from the 384 Caucasian Americans in the deLiver cohort**. We surveyed nine QS scores from 0.1 to 0.9 at 0.1 steps. At each QS score, we computed the faction of SNPs that passed that score (yield) as well as the imputation accuracy using 1% of the SNPS that were randomly masked. In this case, accuracy is defined as the fraction of imputed genotypes that matched the observed genotypes. For each parameter setting, we conducted the random masking and imputation twice and obtained very similar results from the two realizations. Because the accuracy estimation was derived from a large number of SNPs (N ≈ 3K) and a large number of subjects (N = 384), the estimation is very stable. The left panel shows that the imputation performance was better on the Ilmn650Y data (red) than on the Ilmn317K data (black), and that untyped SNPs in weak LD with the assayed SNPs were imputed less successfully (black dotted line). The middle and right panels show that the imputation accuracy and yield were higher for the Ilmn650Y data compared to the Ilmn317K data.

We conducted GWI on 384 deLiver study Caucasian American subjects (see materials and methods), where untyped HapMap markers were imputed based on the HapMap CEU reference [6,7,16]. For each untyped SNP in the HapMap marker set, MACH output both predicted genotypes and a quality score (QS). The majority of the imputed SNPs had a high QS. For example, 88% of the untyped SNPs had a QS ≥	 0.8 (Figure 1, left panel, red dashed line). When we conducted imputation only with SNPs on the Ilmn317K array, considerably lower quality scores obtained, suggesting that GWI benefits from the increased coverage achieved with the higher density arrays (more assayed SNPs to begin with). The Ilmn317K array is a tag SNP array developed to maximize genetic coverage. To construct the Ilmn650Y array, additional tag SNPs were appended to the Ilmn317K set. By design, these additional tag SNPs have weaker average correlations (LD) with SNPs in the Ilmn317K set. Consequently, imputations based on the Ilmn317K were not as accurate (Figure 1, left panel, black dotted line).

To assess the accuracy of the imputed genotypes, we randomly selected 1% SNPs from the Ilmn317K panel and set all patients' genotypes on these 1% SNPs as "untyped" (in other words, we masked out these 1% SNPs). Afterwards we imputed the corresponding genotypes for these SNPs using the remaining SNPs in the Ilmn317K or Ilmn650Y sets, respectively (Figure 1, middle panel). We found the 1% random masking had minor impact on imputation performance (Additional file 2). Comparing the imputed and observed genotypes for the 1% masked SNPs, we found the GWI accuracy was very high. At a QS = 0.8, imputations based on the Ilmn650Y genotypes achieved an accuracy of 97.7%. Interestingly, even at the same QS threshold, imputations based on the Ilmn317K genotypes were less accurate than those based on the Ilmn650Y genotypes. Using the masked SNPs, we estimated that 89% of the untyped HapMap SNPs could be imputed with 98% accuracy with the Ilmn650Y array (Figure 1, right panel). Because the accuracy estimation is based on a large number of comparisons (N = 384 subjects × 3,000 SNP ≈ 1E6), the estimation is very stable and exhibits little variance.

**Figure 2:**
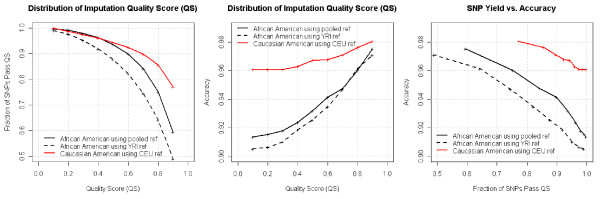
**Genome-wide imputation based on the Affx 500 K plus 164 K custom array data of 200 Caucasian Americans and 200 African Americans from the CATIE cohort**. The left panel shows Caucasian outperformed the African Americans. Also, pooled reference (HapMap YRI+CEU) greatly improved imputation comparing to using YRI reference alone. The middle and right panels shows African American's imputation accuracy was also boosted by pooled reference, although still trailing behind Caucasian counterparts.

In addition to the deLiver cohort, we ran GWI on 400 CATIE subjects. The HapMap CEU and YRI were used as the reference sets for the Caucasian and African American individuals, respectively. For the Caucasian Americans in this cohort, imputations based on the Affx500K + custom array genotypes gave performance better than Ilmn317K, but worse than Ilmn650Y in the deLiver cohort (Figure 2, left panel). Given the relatively weak inter-marker LD in African Americans (compared to Caucasian Americans) as well as the potential population admixture (Additional file 3), GWI on African American samples resulted in a considerable reduction in accuracy (compared to results achieved in the Caucasian American samples). The Eigenstrat method [17] detected an admixture of European and African genetic components in the African American subjects (see materials and methods). Given this type of admixture, it is natural to use a pooled reference (HapMap CEU + YRI) for the GWI. We found the pooled reference greatly boosted GWI performance in African Americans, although still below the Caucasian American samples (Figure 2, middle and right panels). This finding is consistent with the report of Guan et al, who pointed out that the GWI accuracy could be relatively robust as long as the reference panel contained at least some individuals with genetic variation representative of the study cohort [12]. Among the African Americans in the CATIE set, 75% of the untyped SNPs in the HapMap set were imputed with 96% accuracy using the pooled reference. In contrast, all HapMap SNPs can be imputed at this accuracy level in Caucasian Americans.

### The statistical power of imputation-based association tests

Imputed genotypes may be useful in a variety of downstream analyses. One notable application is in testing for association with phenotypes of interest. Perhaps the most important question in this context is whether incorporating GWI genotypes enhances the statistical power to detect associations compared to the power achieved using only the assayed SNPs. Previous studies have used theoretical arguments and simulation studies to address this issue [5,18]. Conditioning on a number of assumptions, GWI was found to increase power moderately over that achieved by the Affx500K set. With the ~40,000 gene expression traits scored in the deLiver cohort, we attempted to quantify the power to detect expression quantitative trait loci (eQTL) using GWI. The large number of phenotypes scored in this cohort provides a path to estimate the power empirically, reducing the dependency on theoretical arguments and simulations where underlying assumptions may not precisely hold.

For the power comparisons, we focused only on cis eQTLs [19](i.e., associations in which the structural gene corresponding to the expression trait and the associated SNP are within 1 million base pairs), given the sample size was too small to capture a significant proportion of the trans eQTLs (i.e., structural gene corresponding to the expression trait and the associated SNP are more than 1 million base pairs away or are located on different chromosomes)[15]. This strategy has been previously applied to benchmark statistical power of SNP arrays in GWAS [3]. In brief, at a fixed false discovery rate (FDR), the number of cis eQTLs detected by a given method can be interpreted as the relative statistical power in the GWAS for the method. The underlying rationale is straightforward. In real data, although we could not determine whether a particular discovery was true or false, at a give FDR (e.g. 10%) we know the proportion (e.g. 90%) of discoveries that are true. Therefore, at a fixed FDR, when two methods result in a different number of discoveries (termed as N_1 _and N_2_) there would be (1-FDR)*N_1 _and (1-FDR)*N_2 _true findings and N_1_/N_2 _is proportional to the relative power of the two methods. Towards that end, single-marker Kruskal-Wallis association tests were conducted to detect the *cis *eQTL for each of the ~40,000 gene expression traits profiled in the deLiver cohort. To empirically estimate the FDR we repeated these tests on permuted gene expression data sets. In each permutation run, we first randomized the patient IDs in the expression file, breaking any association between expression traits and genotypes while leaving the respective correlation structures among gene expression traits and SNP genotypes intact. Then we repeated the association tests for every expression trait and genotype pair in the permuted sets, leading to a set of null statistics for each permutation. A standard FDR estimator was then applied to the resulting association statistics, as previously carried out on observed and permutation null statistics [20].

**Figure 3:**
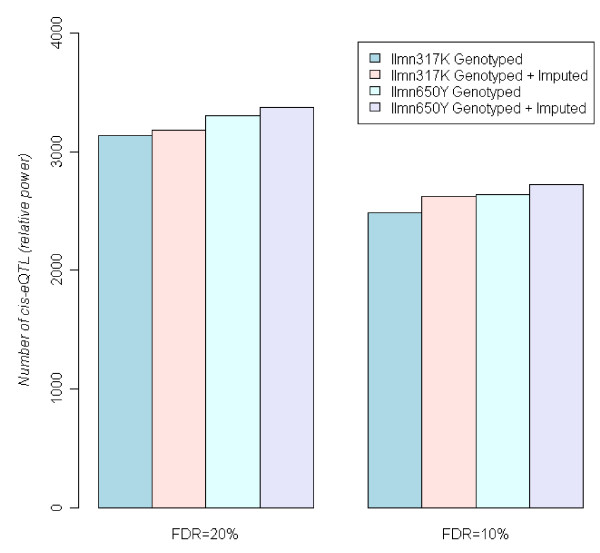
**At fixed FDR, the number of cis eQTLs reflects the relative power of different association study methods**. We found imputation provided modest extra power on top of Ilmn317K and Ilmn650Y.

We compared four different strategies in GWAS of gene expression phenotypes: (1) directly testing for associations using the Ilmn317K SNPs, (2) testing for associations using the entire imputed HapMap SNP set based on the Ilmn317K genotype data; (3) directly testing for associations using the Ilmn650Y SNPs; and (4) testing for associations using the entire imputed HapMap SNP set based on Ilmn650Y genotype data. After the imputation step we filtered out SNPs with a low QS (i.e., QS < QS_cutoff_). We found that the statistical power of the GWAS on the gene expression traits was insensitive to the QS_cutoff_, where QS_cuttoff _∈ [0.1, 0.9] gave similar results (Additional file 4). Figure 3 highlights the number of cis eQTLs (interpreted as relative power) at a QS_cuttoff _= 0.3 according to the suggestion of the Mach's authors. Interestingly, GWI only modestly improved power (by 5.5% and 3.3% for Ilmn317K and Ilmn650Y, respectively) comparing to the analyses with assayed SNPs only. It should be noted that the Ilmn317K + GWI offer less power than the Ilmn650Y itself, suggesting higher density arrays cannot be totally replaced by imputation.

### Resolution of imputation-based association peaks

Because imputation improves the power to detect eQTL, we explored whether the significance levels for cis eQTL p values were significantly improved and whether such improvements enhanced the resolution of the association peaks. In the deLiver cohort genotyped on the Ilmn650Y array, there is only a modest improvement in power (3.3%) to detect cis eQTL. That is, almost all of the cis eQTL identified in the GWI data set were detected when only assayed SNPs were looked at. However, we found that 43.2% of the cis eQTL got smaller p-values when incorporating imputation compared to the results using assayed SNPs only. The significance levels for roughly 10% and 4% of the cis eQTLs improved by more than one and two orders of magnitude, respectively. If in these cases the GWI data were providing SNPs that were in stronger LD with the causal variants, we would expect that for the cis eQTL the most significantly associated SNPs would be found closer to the structural gene. This in fact was exactly what we found.

**Figure 4:**
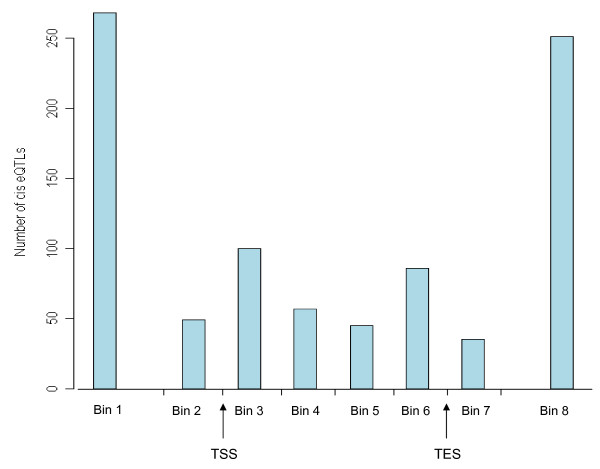
**To study the location of cis eQTL peaks, we divided each gene into eight bins using the quarter-length of that gene as the unit**. For example, bin#1 denotes the region of a gene that is more than 1 unit upstream of the transcription start site (TSS), and bin#2 denotes the region of the gene that is immediately upstream of the TSS. Here we restricted attention to the most highly significant cis eQTLs (those with p ≤ 1E-10) and found the number of peaks (the best hit SNP in the eQTL) in bin#1 through bin#8 were 268, 49, 100, 57, 45, 86, 35, and 251, respectively. A bimodal distribution is apparent in this plot. We surveyed additional p-value cutoffs in addition to 1E-10, and observed consistent results in which the middle part of the genes harbored less cis eQTLs than the TSS and TES regions.

We classified cis eQTL detected by Ilmn650Y assayed SNPs into two categories: (1) transcriptional start site (TSS) eQTL in which the QTL peak was closer to the TSS of the gene than to the transcriptional end site (TES); and (2) TES eQTL in which the eQTL peak was closer to the TES of the gene than to the TSS. For TSS eQTL the overall median distance of the eQTL peak to the TSS moved from -13.8 Kb to -9.9 Kb by incorporating imputation, where the negative distance implies the eQTLs clustered just upstream of the TSS. More striking were the TSS eQTLs whose significance level gained at least one order of magnitude. In this case the median distance to the TSS shifted from -19.4 Kb to +1.6 Kb. For TSS QTLs whose significance level gained at least two orders of magnitude, the median distance to the TSS shifted from -19.8 Kb to +1.0 Kb. For TES eQTLs, the overall median distance of the eQTL peak to the TES moved from +15.0 Kb to +7.3 Kb by incorporating imputation, where the positive sign implies the eQTLs clustered just downstream of the TES. For TES QTLs whose significance level gained at least one order of magnitude, the median distance to the TES moved from +17.8 Kb to -0.3 Kb. For TES QTLs whose significance level gained at least two orders of magnitude, the median distance to the TES moved from +25.9 Kb to -0.8 Kb. Figure 4 makes it clear that the location of the cis eQTL peaks form a bimodal distribution around the TSS and TES sites, with the middle regions of the genes having fewer cis eQTL than the regions surrounding the TSS and TES sites. From another viewpoint, we saw the strong association hits resided close to the structural gene (Additional file 5).

## Discussions

The genome-wide imputation of genotypes has recently attracted significant attention given its broad applicability in the era of GWAS. It is likely that many disease variants have small effects, so that even today's large studies are underpowered to detect most of these effects. Therefore, combining data across multiple studies will be essential to uncovering the genetic complexity of common human diseases. Given this, one of the major utilities of genotype imputation is in combining data from studies that use different genotyping chips to facilitate the meta analysis of multiple GWAS [5]. We explored three critical issues related to GWI: (1) accuracy of the imputed genotypes, (2) the extent to which imputation increases the power to detect associations, and (3) degree to which imputation increases resolution of the association peak.

The first issue we explored is particularly relevant for combining datasets. We systematically assessed a number of factors that had the potential to influence the accuracy of imputed genotypes. First, because accuracy depends on SNP density, we observed that the Ilmn650Y was superior to earlier versions of arrays. Further, we expect the new Affymetrix SNP 6.0 will offer good imputation performance similar to Ilmn650Y because their genetic coverages are comparable. Hence, the price of the array could be the most important factor in choosing a genotyping platform, given sample size has a profound impact on statistical power [3]. Second, the similarity of LD patterns between the study sample and the reference has a significant impact on accuracy given the untyped SNPs are imputed based on haplotypes seen in the reference population (e.g. HapMap samples). Our findings indicate the need to extend the HapMap project to additional ethnic groups. For example, extending HapMap data on Native Americans would be useful for GWI in Hispanic American subjects. Third, we found high accuracy could not be achieved when the untyped SNPs were in weak LD with assayed ones. For example, untyped Ilmn650Y SNPs were imputed less successfully by Ilmn317K than randomly selected HapMap SNPs. This finding is theoretically easy to understand and nicely consistent with simulation studies, where Pei et al found all imputation methods performed better in strong LD regions versus weak LD regions [13]. In addition, we observed low GWI accuracy in African Americans compared to Caucasian Americans, given the genetic coverage of the SNP arrays were lower in African populations. This is related to the dependency of LD strength on imputation accuracy, given the relatively weak LD for African subjects [21]. Zhao et al recently reported the IMPUTE software achieved substantially higher accuracy in Caucasian and Asian subject compare to African subjects, a difference explained by their LD differences [21]. Arrays with even higher marker density are necessary to capture more genetic information in genomes of African subjects, and such arrays will boost GWI performance in African American samples. Fourth, when studying GWI accuracy by randomly masking out SNPs, we also binned masked SNPs into minor allele frequency (MAF) categories (Additional file 6) and examined whether MAF affected accuracy. At high QS scores (e.g. QS ≥ 0.8), MAF had little impact. At low QS scores, we saw significantly more errors for high frequency SNPs. These results are consistent with those of Pei et al, where they found MAF affected accuracy in low but not high LD regions [13]. Finally, population structure could affect GWI accuracy as well as bias downstream association tests. We found eigenstrat and the human genetics diversity project (HGDP) were highly powered for detecting population admixture, which have been shown in previous reports [17,22-25]. More importantly, application of these tools to our study samples revealed relationships to the 51 ethnic groups collected worldwide, guiding the appropriate choice of GWI reference. For example, HGDP elucidated the admixture of the Native American and Caucasian genetic components in Hispanic American samples. Therefore, including Native American data in the GWI reference panel would be critical to achieving high accuracy when imputing Hispanic American subjects. One caveat should be noted, while GWI can be successfully carried out in the presence of population admixture, such admixture could nevertheless lead to false-positive associations unless proper adjustments are made.

The second issue we explored in the context of GWI was statistical power, one of the most critical issues in genetic studies involving complex traits. Whether GWI genotypes provide extra power in a GWAS setting has been studied via simulation [4,5,18]. However, such studies make a number of modeling assumptions that may or may not be true in practice. By leveraging the ~40,000 expression phenotypes measured in the liver gene expression cohort [15], we were able to assess statistical power empirically. Incorporating GWI provided a 5.5% increase in power with respect to the Ilmn317K array. This increase in power is likely due to the incomplete coverage by the Ilmn317K array, so that GWI is able to extract moderately more information from the genome. Similar results were obtained for the Affx500K array [5]. In contrast, the power gain by GWI is only 3.3% over that achieved by the Ilmn650Y array, given the already high genetic coverage by this array. Taken together, the power increase achieved by imputing genotypes is not more dramatic because the SNP arrays considered in this study are already quite dense, in addition, imputation introduces more tests, resulting in an increase in the multiple testing penalty. Interestingly, the power of the Ilmn317K SNPs + Imputed SNPs resulted in lower power than the Ilmn650Y SNPs to detect cis eQTL in the deLiver cohort (Figure 3), indicating the regions poorly covered by the Ilmn317K SNPs cannot be recovered by imputation. That is, the latest high density arrays cannot simply be replaced by GWI even for studies on Caucasians. These observations are consistent with previous reports that there was not a substantial gain in power by genotyping all common SNPs compared to genotyping only those SNPs represented on the Ilmn650Y array [3]. The Ilmn650Y (Affymetrix SNP 6.0 as well) are tag SNP arrays trained on the HapMap data (270 individuals and about 2.5 million common SNPs). Hence, GWI using HapMap data as a reference will not provide much additional information. Given 7.5 million common SNPs exist in the human genome [1], it is essential to generate a reference SNP panel that goes beyond HapMap (e.g., incorporating novel SNPs and recruiting a greater diversity of individuals) if we hope to significantly increase the power gains that can be achieved by GWI in GWAS.

Finally, we found that GWI provided a denser association map with superior resolution power, enhancing our ability to define the boundaries of the association peak and infer the true causal variants. While the majority of cis eQTLs could be identified using only assayed SNPs, we found that imputation enhanced the p-values for 43.2% of the cis eQTLs. More importantly, incorporating GWI shifted the QTL peaks (i.e., the smallest p-value in the QTL) closer to the structural genes. For example, the TSS eQTLs, whose significance level gained at least one order of magnitude by imputation, moved considerably closer to the TSS (median distance shifted from -19.4 Kb to +1.6 Kb with imputation). The strongest eQTLs tend to cluster near the genes' TSS and TES regions, forming a bimodal distribution (Figure 4). These observations match our current understanding that transcription initiation driven at the TSS of the gene is among the most important determinants of transcript levels, and supports a growing number of observations from the ENCODE project and others that many transcription factors bind near the TES of the gene. In addition, miRNAs are known to affect transcript stability and often bind transcript regions that are near the TES. Given the above considerations, we believe the shift of QTL peaks when incorporating GWI indicates the association hits are more proximal to the causal variants.

Multiple testing is a critical issue for GWAS with or without incorporating GWI genotypes. In the paper, we did not focus on the SNP-trait association p-value, because it certainly requires rigorous correction. Instead, we derived FDR empirically, which addressed the multiple testing and allowed direct comparison of number of discoveries (i.e., relative statistical power) among various SNP panels. The Bayes factor measures the impact of the data on the support for H_o _in preference to H_a_. It has been used in an eQTL study by Veyrieras et al [26]. The interpretation of a Bayes factor obviates the need for an adjustment for multiple comparisons. The frequentist and Bayesian approaches have been compared on simulated data [12], where the two strategies performed similarly at low FDRs (e.g. 10%). However, it is difficult to compute the FDR using Bayes factors on real data where the truths are unknown. In contrast, FDR is straightforward to derive using frequentist methods. As discussed above, the FDR provides a path to empirically assess statistical power, and so we chose the frequentist approach for the analyses carried out herein.

Clearly, GWI is very accurate when based on genotypes of the today's high density arrays. Previously we proposed methods to incorporate genotype uncertainty in association test [27], and found that low genotype error rates (e.g. 2%) had almost no impact on power or point estimation of effect size. Therefore, conventional test methods might be sufficient. The MACH algorithm outputs the QS for each SNP, which provides a path to control the imputation uncertainty. Shown in Additional file 4, we chose different QS cutoffs to filter out less accurately imputed SNPs and found the statistical power was not sensitive to the filtering. In summary, we found the Ilmn650Y and Affx500K + custom array could impute the entire HapMap set accurately, at least among Caucasians. This is encouraging news for researchers regarding merging data created on different platforms. Our results may also serve as a guide with respect to choosing an array type for a given study. Because sample size has a more profound impact on GWAS statistical power compared to genetic coverage of the SNP array [3], genotyping more subjects using cheaper arrays will provide significantly more power to detect associations between SNPs and traits of interest.

## Conclusion

Current algorithms can accurately impute genotypes for untyped markers, which enables researchers to pool data between studies conducted using different SNP sets. While genotyping itself results in a small error rate (e.g. 0.5%), imputing genotypes is surprisingly accurate. We found that dense marker sets (e.g. Ilmn650Y) outperform sparser ones (e.g. Ilmn317K) in terms of imputation yield and accuracy. We also noticed it was harder to impute genotypes for African American samples, partially due to population admixture, although using a pooled reference boosts performance. Interestingly, GWAS carried out using imputed genotypes only slightly increased power on top of assayed SNPs. The reason is likely due to adding more markers via imputation only results in modest gain in genetic coverage, but worsens the multiple testing penalties. Furthermore, cis-eQTL mapping using dense SNP set derived from imputation achieves great resolution, and locate associate peak closer to causal variants than conventional approach.

## Methods

### HapMap Reference Panel

The International HapMap data comprised 270 individuals from four ethnic groups: (i) 30 trios from the Yoruba, in Ibadan, Nigeria (YRI); (ii) 30 trios from the CEPH collection (Utah residents with ancestry from Northern and Western Europe) (CEU); (iii) 45 unrelated Han Chinese individuals from Beijing, China (CHB); and (iv) 45 unrelated individuals from Tokyo, Japan (JPT). We downloaded the phased CEU and YRI haplotypes of HapMap release 22 as GWI reference panel.

### deLiver study subjects

Liver tissue samples were collected from "Liver Study subjects", whose detailed characteristics were reported in a separate article [15]. It was a joint effort of three independent institutes, Vanderbilt University, the University of Pittsburgh, and Merck Research Laboratories. All samples and patient data were handled in accordance with the policies and procedures of the participating organizations. DNA specimens were extracted and sent to Illumina Inc. for genotyping service using Ilmn650Y. Additionally, we purified RNA from the tissue samples and measured the approximately 40,000 gene transcription levels using the Agilent platform. In total, 384 Caucasian subjects with known gender were successfully mRNA profiled and SNP genotyped. Furthermore, we filtered out SNPs with call rate < 90%, and totally 574 K autosomal SNPs were used in the analysis. Illumina 317 K array (Ilmn317K) contains a subset of Ilmn650Y's SNPs, therefore, we also derived Ilmn317K data (contain 300,854 SNPs after quality control) for deLiver samples.

In addition, DNA specimens were collected from eight African American liver donors, and underwent Ilmn650Y genotyping. Due to the small sample size, we did not run GWI or WGAS on these eight subjects, but use them to illustrate population admixture among African Americans.

### CATIE subjects

We selected 200 Caucasian American and 200 African American from CATIE cohort, based on self reported ethnicity [14]. CATIE was a multiphase randomized controlled trial of antipsychotic medications involving schizophrenia patients. All cases were participants in the CATIE project, which was conducted between January 2001 and December 2004. Individual genotyping was conducted by Perlegen Sciences using Affymetrix 500 K chipset and a custom 164 K chip created by Perlegen, and made public available. Rigorous quality control steps removed 157,048 SNPs, and the remaining 492,900 SNPs entered the analysis [14].

### Human Genetics Diversity Project (HGDP)

938 unrelated individuals from 51 populations (collected in Europe, Middle East, Central/South Asia, Africa, East Asia, America and Oceania) of the HGDP were successfully genotyped using Ilmn650Y [28], and data has been made available to the public. Principal components (PCs) built on over 600 K assayed SNPs provide high resolution to separate subjects from different continents. We implemented the eigenstrat algorithm [17], and derived identical results as Li et al [28]. Further, we projected the deLiver subjects to PC space defined by HGDP data (termed as HGDP-PC space) and examined population admixture in our samples. The Caucasian Americans clustered tightly and collocated with HGDP Europeans. However, the eight African Americans show certain degree of admixture, in another word, deviation from the HGDP African populations towards the HGDP European cluster. Such results suggest European genetic components in African American samples.

At the current stage, HGDP has not been typed on Affymetrix arrays, which only shares a small number of SNPs with Ilmn650Y. Therefore, we are not able to build HGDP-PC space on Affx500K SNPs and did not project CATIE individuals.

### Association Test

Kruskal-Wallis (KW) one-way analysis of variance was employed in testing association between gene expression traits and genotypes. The KW test can be considered as the non-parametric counterpart to ANOVA for testing equality among groups (e.g., the three genotype groups corresponding to a given SNP). This test does not assume the traits are normally distribute and therefore is more robust to outliers and violations of other assumptions important for successful application of parametric tests. In brief, the KW test was applied on a given trait-SNP pair by first ranking all trait values regardless of genotype, assigning tied values the average of the ranks they would have received had they not been tied. Then we computed the test statistic (K) as



where n_i _is the number of subjects for genotype i; r_ij _is the rank of subject j who carried genotype i; N is the entire sample size; and g denotes the number of genotype groups (either 2 or 3 for the groups tested). Finally, the p value was derived using the approximation Pr(χ^2^_g-1 _≥ K).

## Authors' contributions

KH and EES designed the study. KH, EC and JM prepared the data sets and conducted the analysis. KH and EES wrote the manuscript. All authors read and approved the final manuscript.

## Supplementary Material

Additional file 1 **Figure S1**. Beagle and Mach showed similar accuracy in imputing missing genotypes. Although we did not apply quality score (QS) filtering, Mach results were still slightly better. We noticed relatively low SNP density on chromosome 19, where imputation was consequently less accurate. Click here for file

Additional file 2 **Figure S2**. Randomly masking out three thousand SNPs on the array had a minor impact on imputation performance. Click here for file

Additional file 3 **Figure S3**. We used the Ilmn650Y data of the human genetics diversity project (HGDP) samples to constructs principal components (HGDP-PCs), and projected eight African American (termed as "deLiver AfrA" in the figure) samples onto this HGDP-PCs space. Although located close to the African groups, these eight subjects shifted towards the European cluster, indicating population admixture. Click here for file

Additional file 4 **Figure S4**. Choosing the QS cutoff had little impact on the power of eQTL mapping. In this paper, we used QS ≥ 0.3, according to the suggestions of the software's authors. Click here for file

Additional file 5 **Figure S5**. We classified the cis eQTLs detected using the Ilmn650Y set into two categories: (1) QTL whose best hit was closest to the transcription start site (TSS) of the gene relative to the transcriptional end site (TES; panel A); and (2) QTL whose best hit was closest to the TES relative to the TSS (panel B). Clearly, the SNPs that strongly associated with mRNA expression levels were clustered around the TSS and TES regions of the genes. Click here for file

Additional file 6 **Figure S6**. SNP minor allele frequency has only a small impact on imputation accuracy. Click here for file
