# Supplementary figures and images for: Accuracy of genome-wide imputation of untyped markers and impacts on statistical power for association studies
Source: BMC Genet. 2009 Jun 16;10:27. doi: 10.1186/1471-2156-10-27 (PMC2709633; doi:10.1186/1471-2156-10-27)

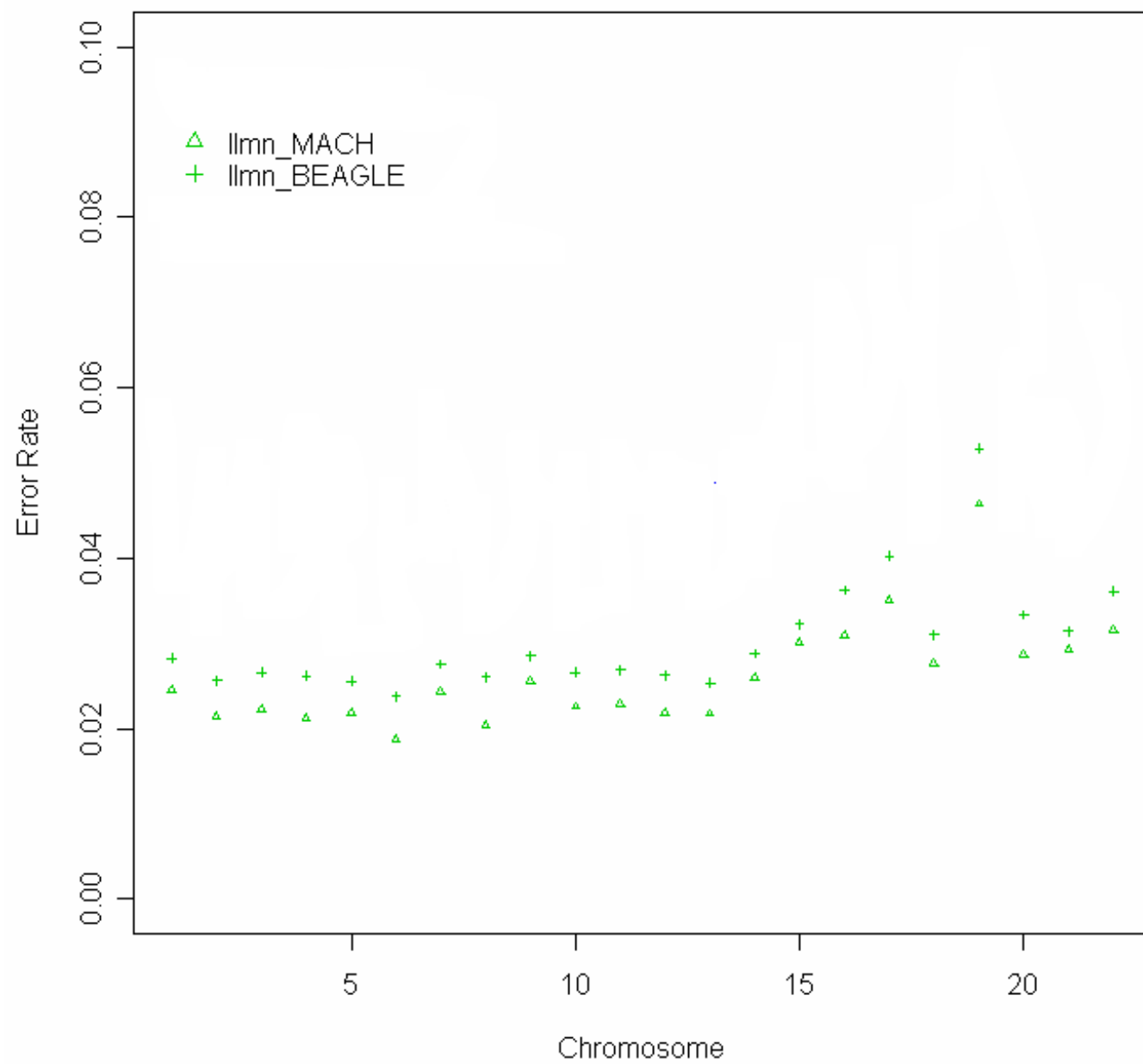

Supplement: Additional file 1 — Figure S1. Beagle and Mach showed similar accuracy in imputing missing genotypes. Although we did not apply quality score (QS) filtering, Mach results were still slightly better. We noticed relatively low SNP density on chromosome 19, where imputation was consequently less accurate. [file 1471-2156-10-27-S1.pdf]

**Distribution of Imputation Quality Score (QS)**

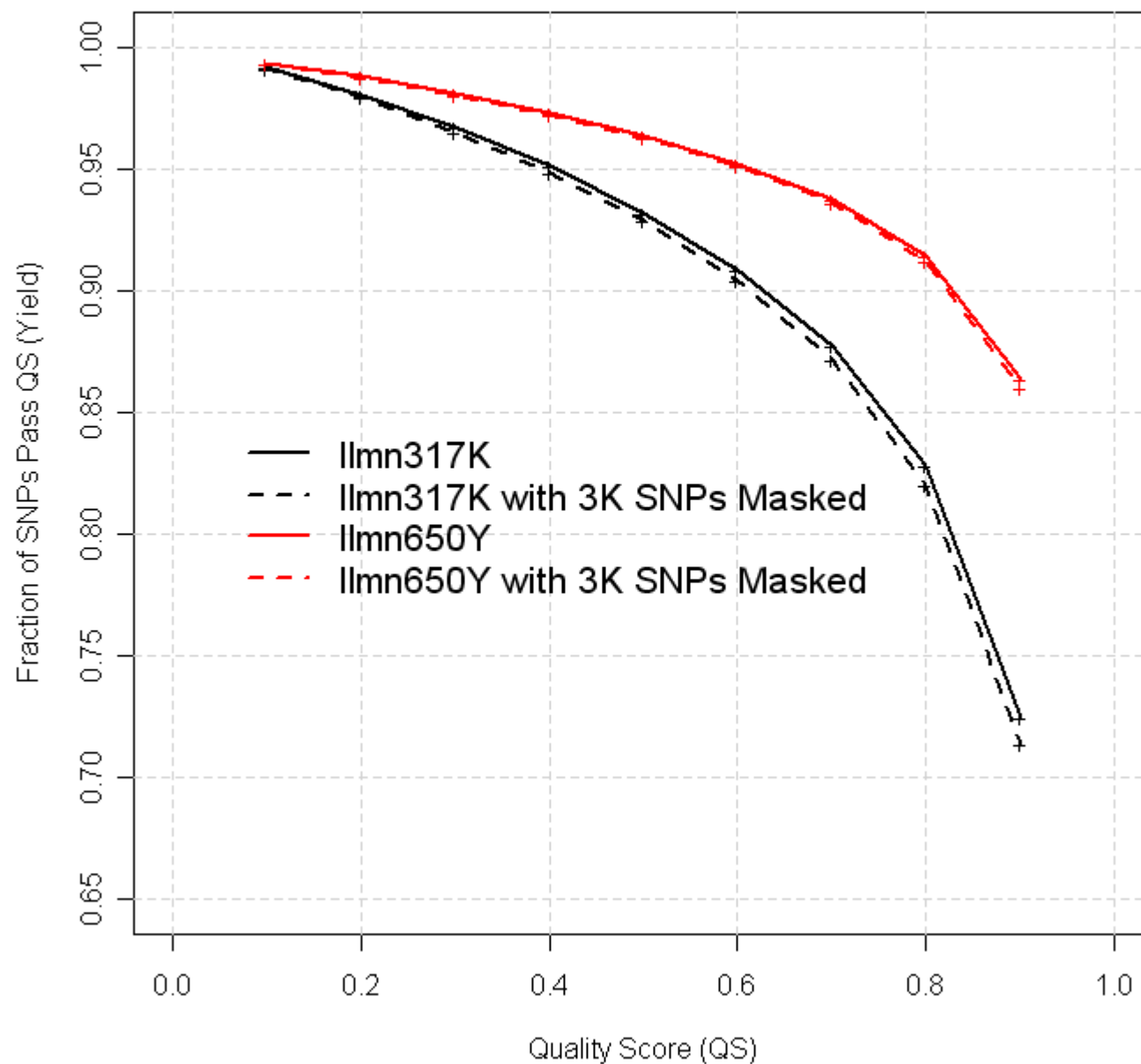

Supplement: Additional file 2 — Figure S2. Randomly masking out three thousand SNPs on the array had a minor impact on imputation performance. [file 1471-2156-10-27-S2.pdf]

Figure S3

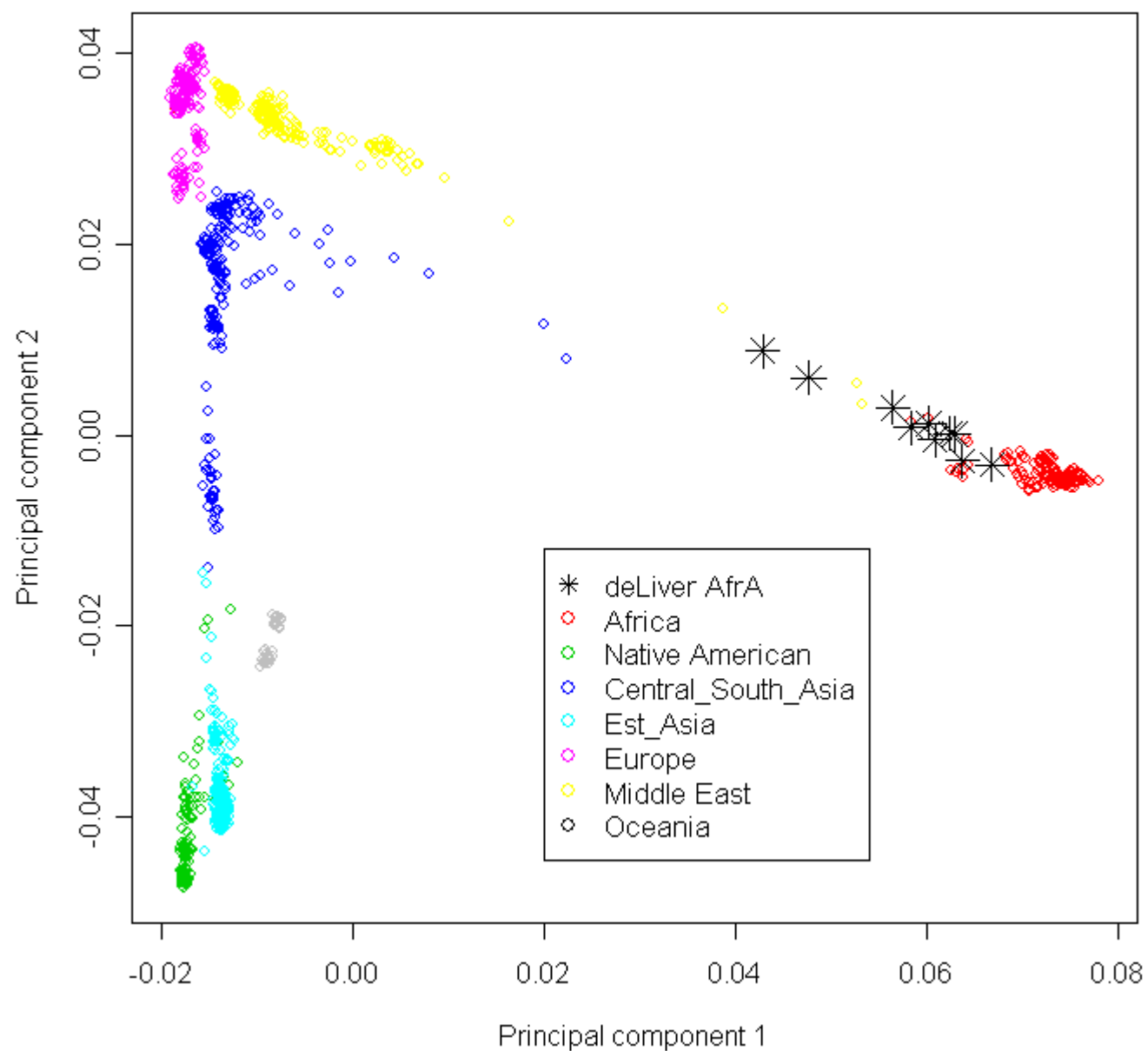

Supplement: Additional file 3 — Figure S3. We used the Ilmn650Y data of the human genetics diversity project (HGDP) samples to constructs principal components (HGDP-PCs), and projected eight African American (termed as "deLiver AfrA" in the figure) samples onto this HGDP-PCs space. Although located close to the African groups, these eight subjects shifted towards the European cluster, indicating population admixture. [file 1471-2156-10-27-S3.pdf]

A

Ilmn317K

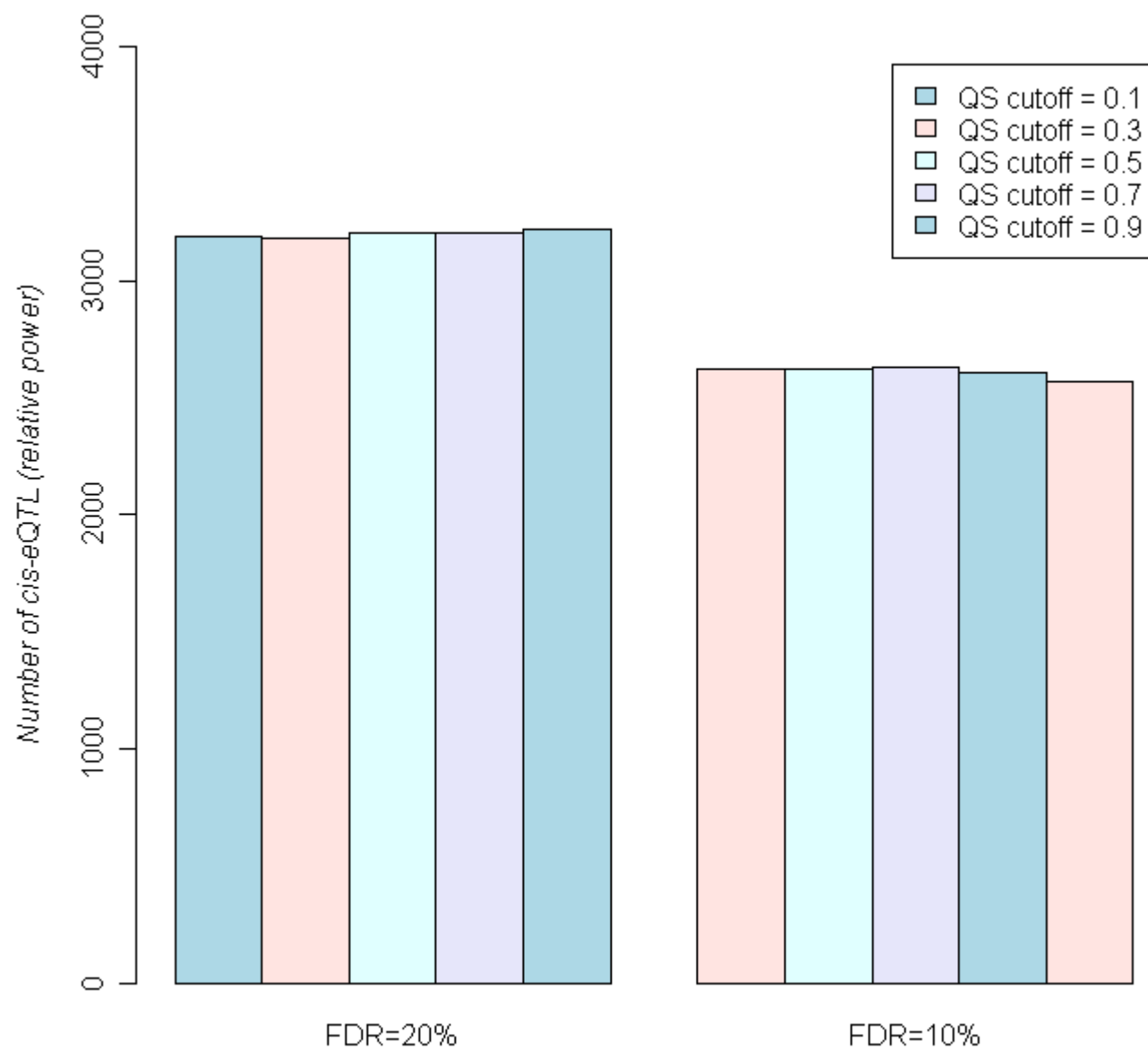

**B****Ilmn650Y**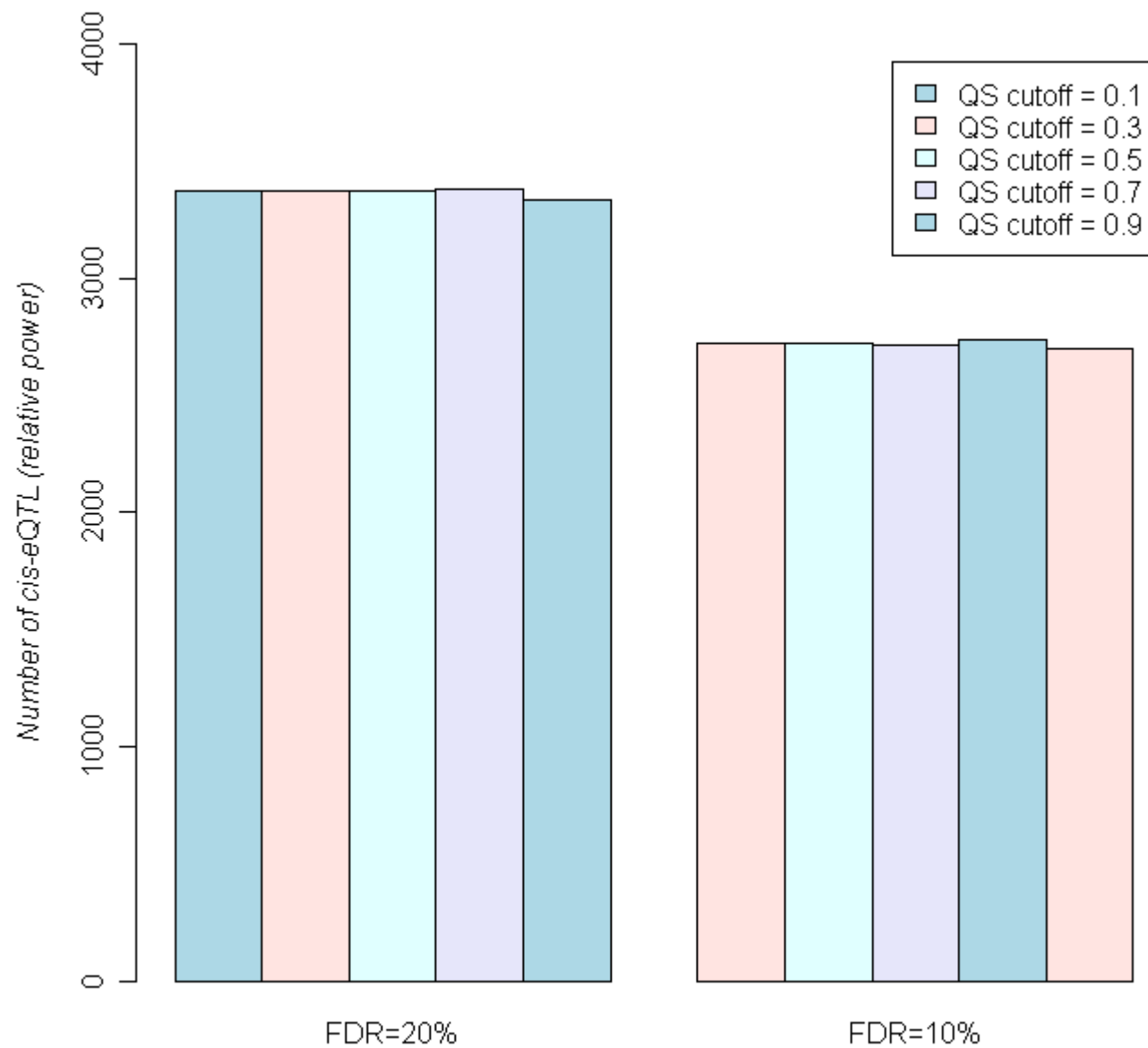

Supplement: Additional file 4 — Figure S4. Choosing the QS cutoff had little impact on the power of eQTL mapping. In this paper, we used QS ≥ 0.3, according to the suggestions of the software's authors. [file 1471-2156-10-27-S4.pdf]

A

# TSS eQTL

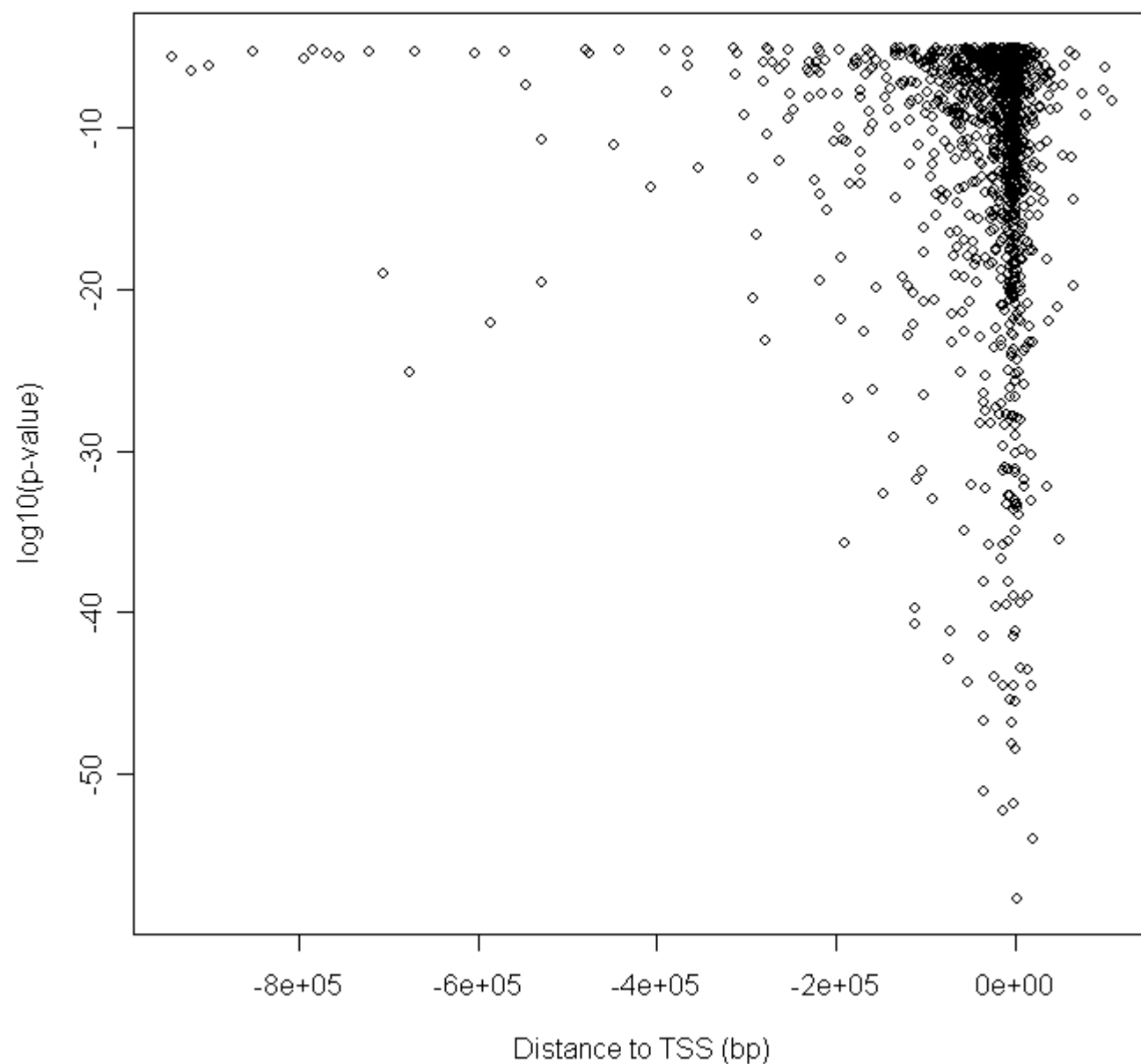

**B****TES eQTL**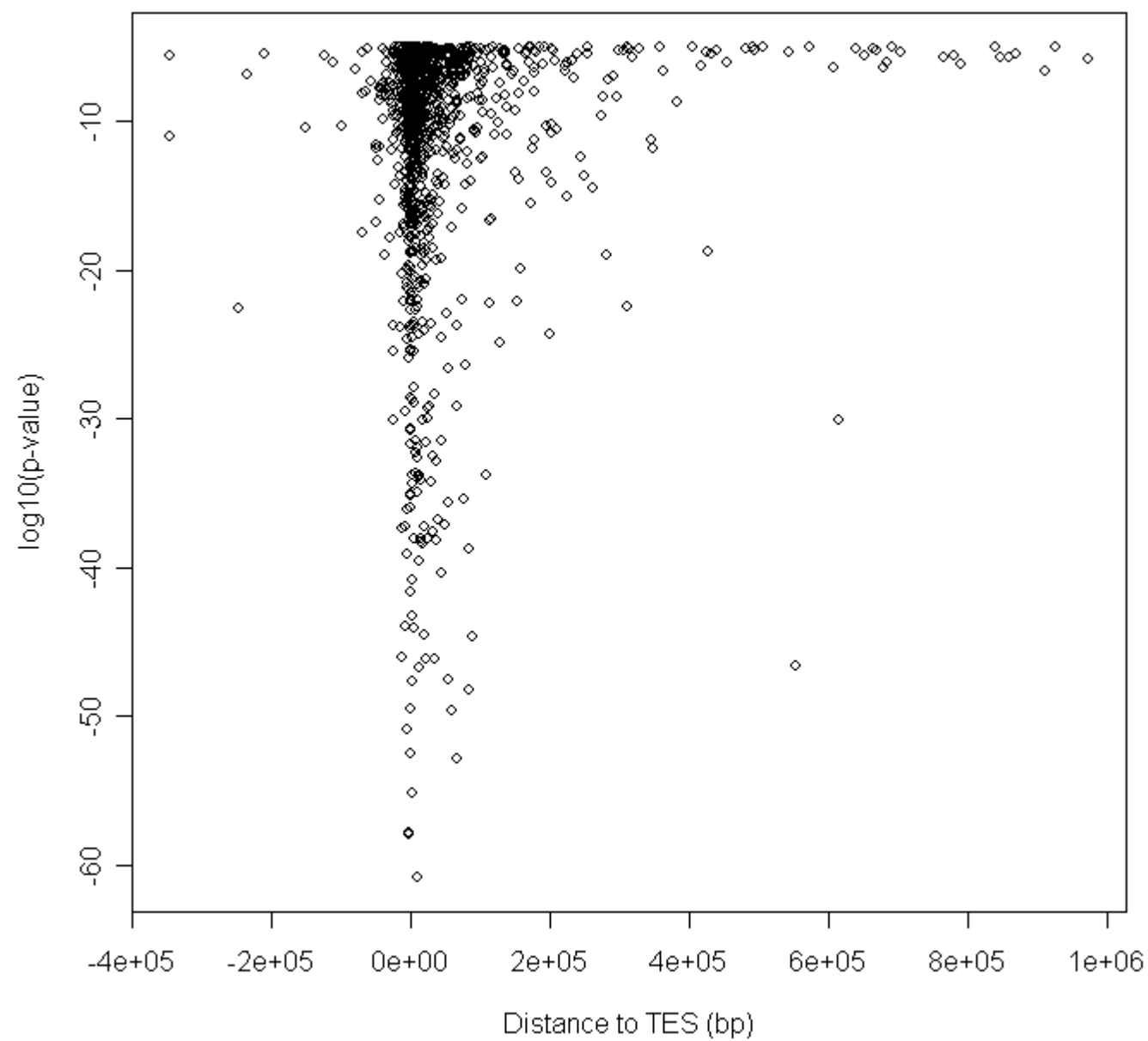

Supplement: Additional file 5 — Figure S5. We classified the cis eQTLs detected using the Ilmn650Y set into two categories: (1) QTL whose best hit was closest to the transcription start site (TSS) of the gene relative to the transcriptional end site (TES; panel A); and (2) QTL whose best hit was closest to the TES relative to the TSS (panel B). Clearly, the SNPs that strongly associated with mRNA expression levels were clustered around the TSS and TES regions of the genes. [file 1471-2156-10-27-S5.pdf]

**Impact of SNP MAF on Imputation Accuracy, Ilmn317K**

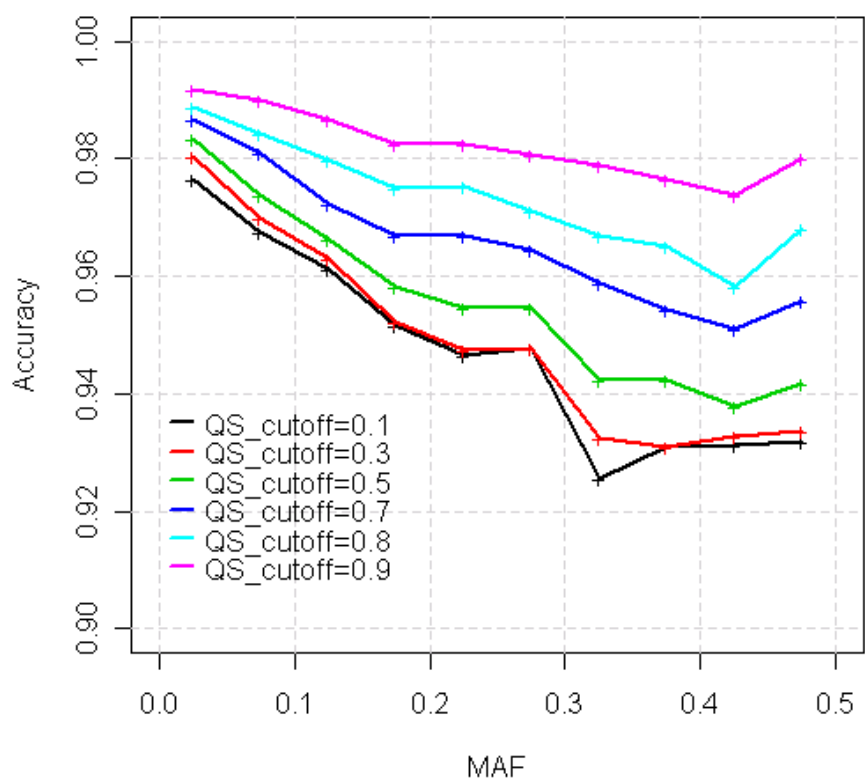

**Impact of SNP MAF on Imputation Accuracy, Ilmn650Y**

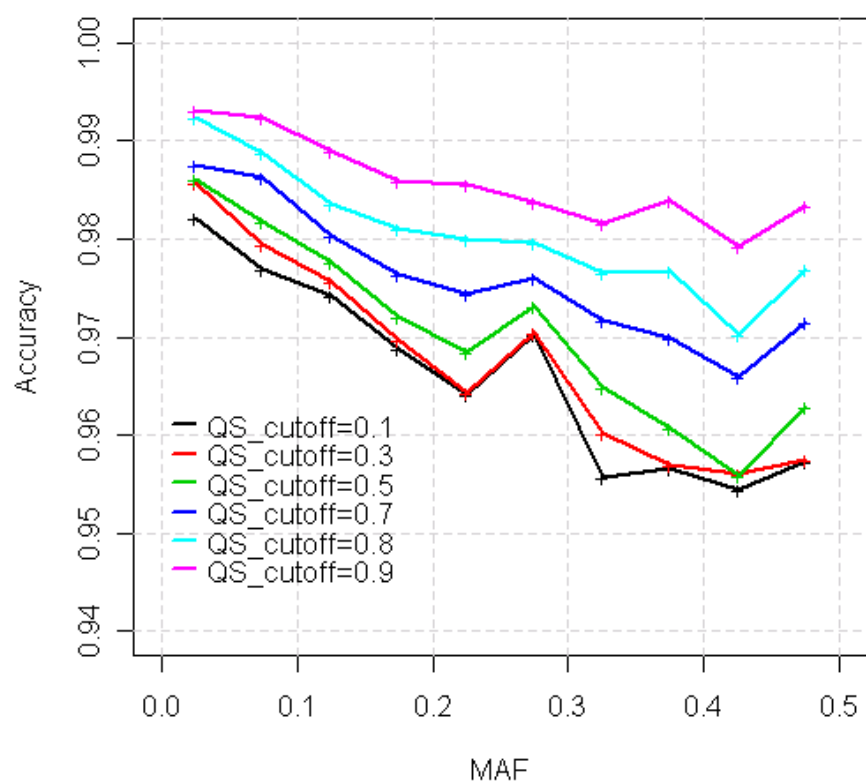

Supplement: Additional file 6 — Figure S6. SNP minor allele frequency has only a small impact on imputation accuracy. [file 1471-2156-10-27-S6.pdf]
